# Creative AI and Musicking Robots

**DOI:** 10.3389/frobt.2021.631752

**Published:** 2021-11-24

**Authors:** Craig Vear

**Affiliations:** Institute of Creative Technologies, De Montfort University, Leicester, United Kingdom

**Keywords:** music, robots, creativity, human-centered AI, creative AI

## Abstract

This article discusses the creative and technical approaches in a performative robot project called “*Embodied Musicking Robots*” (2018–present). The core approach of this project is human-centered AI (HC-AI) which focuses on the design, development, and deployment of intelligent systems that cooperate with humans in real time in a “deep and meaningful way.”^1^ This project applies this goal as a central philosophy from which the concepts of creative AI and experiential learning are developed. At the center of this discussion is the articulation of a shift in thinking of what constitutes creative AI and new HC-AI forms of computational learning from inside the flow of the shared experience between robots and humans. The central case study (*EMRv1*) investigates the technical solutions and artistic potential of AI-driven robots co-creating with an improvising human musician (the author) in real time. This project is ongoing, currently at v4, with limited conclusions; other than this, the approach can be felt to be cooperative but requires further investigation.

## Introduction

### The Goal

The aim of this practice-based research project was to investigate the technical solutions and artistic potential of AI-driven robots co-creating with a human musician in real time. This research extends and enhances existing research in this area, specifically that of computational creativity (e.g., McCormack and d’Inverno (McCormack and d’Inverno, 2012)), AI and music (e.g., Miranda (Miranda, 2021)), and robotic musicianship (e.g., Bretan et al. (Weinberg et al., 2020)), with a specific focus on the embodied relationship among agent- robot, sound presence, human musician, and the flow of co-creativity, with the aim of enhancing the creativity of humans. This is a rich and emerging area with many solutions which are currently being developed, most of which are dealing with in-the-loop solutions for human–robot music interaction. For example, the cooperative AI at the heart of “*In A Silent Way*” (McCormack et al., 2019) is trained using performance data and communicates with the human musicians through real-time sound generation and emoticons in order to generate a sense of trust. Additionally, in *Design Considerations for Real-Time Collaboration with Creative Artificial Intelligence*, McCormack (McCormack et al., 2020) offers a framework for maximizing the human–AI creative interaction, which can be migrated to human–robotic musicking.

The *Embodied Musicking Robots* (EMR) project contributes to this discussion by asking the following research question:

If we want robots to join us inside the creative acts of music, then how do we design and develop robot systems that prioritize the relationships that bind musicians inside the flow of music-making?

This question comes from deep and meaningful experiences that I had as a high-level professional musician for over 30 y and support the core goal of HC-AI. As such, its focus is to seek solutions for the stimulation of the relationships generated inside the real-time music-making, which I outlined in detail in Vear (2019) with its basic structure being split into these two domains:

1) *Taking in*: within the flow, musicians make connections with the AI as they reach out, suggest, offer, and shift through the tendrils of affordance experienced through the notions of.
⁃ *Liveness*: the sensation that the AI is cooperating in the real time making of music, and this meaningful engagement feels “alive.”
⁃ *Presence*: an experience that some*thing* is there or I am *there.*
⁃ *Interaction*: the interplay of self with environments, agents, and actants.
2) *Taken into*: the AI can establish a world of creative possibilities for exploration through the flow through the domains of.
⁃ *Play*: the pure play of musicking happens inside a play-sphere in which the idea and musicking are immutably fused.
⁃ *Time*: the perception of time (of now, past, future, and the meanwhile of multiple convolutions of time) inside musicking plays a central role to the experience of the musician
⁃ *Sensation*: is an esthetic awareness in the experience of an environment (music world) as felt through their senses.

However, I must stress that the EMR project is ongoing and so far relatively unfunded, with the limitations imposed by COVID and repeated lockdowns, and has only had the author in-the-loop. Therefore, this article should be read as more a hopeful, position statement, with some (autobiographical) evidence to support the authors understanding that this system feels like it is stimulating relationships inside music-making, rather than simulating the movements and sounds of a robotic musician.

### Definitions

Before I describe the solutions that I designed and deployed in the *EMR* project; I need to simply define what I mean by the following terms within the context of this project. This defining process also helped consolidate the design and development of the Creative AI and robotic systems with the goals of HC-AI.

*Musicking* is the creative acts of real-time music-making. Musicking is a term first created by Christopher Small to define a perspective that “to music is to take part” (Small, 1998). Small wrote that “taking part can happen in any capacity” (Small, 1998) such as performing, composing, and listening (and dancing). It crucially means formation through musicking is formed in the relationships that are established within the realm of taking part with agents, sounds, spaces, and presences that are encountered here.

*Flow* is the experience of musicking from inside the activity. Within the context of this project, the flow of musicking defines how “musicians become absorbed in the music through a sense of incorporation within their environment (the sound world), a shared effort (with the digital, virtual, AI, and robotic agents), and a loss of awareness of their day-to-day wakefulness and bodily self-consciousness (embodiment with their instrument and into their music)” (Vear, 2019).

*Embodiment* (in music) is the process in musicking of drawing the musician’s sound into their bodily sense of being. This presumes that when musicians make music, it is not a process of outputting sound into the world but an embodied experience of becoming the sound they create in the flow of musicking. Equally, it describes the process of the musician reaching out from this sense of becoming and drawing in the sounds of others so they feel their *presence as sound*. This is a dance of sorts: to touch, to feel, to sense, to work with, to play with, and to hide and seek and flirt and subvert with others through the *flow*.

*Creativity*: I recognize creativity when play turns into invention within the flow of musicking. As a musician, creativity has to be of value and meaning to me. It needs to be “greater than the sum of its parts” (Vear, 2019); (Boden, 2003; Zedan et al., 2008; Iacoboni, 2009; Pearce, 2010; Thomsom and Jaque, 2017; Zedan et al., 2017) and go beyond merely creating music (manufacturing sound using one’s skills). It also goes beyond recognizing that something is new, or novel, or that I have innovated in a given situation. Creativity is felt to be fundamentally new—to my mind—and emergent from my playfulness within the flow. It takes effort and needs feeding, and goes beyond “adhering to a list of ingredients and/or instructions within a prescribed situation; emergent creativity—that is, genuinely original—cannot be replicated by simply repeating a set of rules or prescribed circumstances” (Vear, 2019).

Creativity is giving in to a playful situation that *might* return with a creative spark. Creativity is not constant, reliable, or automatic; it needs nurturing with open, generous, and cultivating energy. On the other hand, it can sustain bold and mischievous challenges or seemingly disruptive engagement designed to rail-road ongoing trains of thought, so long as these are still giving in their nature.

In this article, I define three sub-domains of creativity to highlight the human–robot relationships. These are based on my general experience as an improvising musician and are used to identify the types of co-creativity within musicking from the human musician’s perception (note: this project does not deal with notions of machine consciousness or perception):

• *Concurrent*: a sense that both agents (human and robot) are playfully inventing in isolation but within the shared flow of musicking
• *Collaborative*: a sense that both agents are contributing to a shared play idea, feeding a sense of collective invention through individual contribution and perspective
• *Co-creative*: a sense that the robot and human agents are collectively inventing through a stimulated sense that each is in inside the other’s head. By this, I mean that the robot/AI, as perceived by the human musician, is in the loop with the human, and together, they are inventing on a singular idea, feeding each other’s play as if it were one train of thought.

*Creative AI* not only includes practices that have AI embedded into the process of creation but also encompasses novel AI approaches in the realization and experience of such work. I define AI as the design, development, and deployment of intelligent agents that respond with insights from their environment and perform actions. Each agent is mainly concerned with a rational action within a given *situation* [taken from AI a modern approach]. The focus of behavioral and embodied AI emphasizes the close-coupled relationship between the *situation* that an intelligent agent is operating in and the *behavior* that it exhibits to cope inside such a *situation*. As such, the focus on intelligent behavior is on the coping systems that are required to maintain a balance of existing within such a situated environment.

With these definitions in mind, the goal of the *EMR* project is to design and develop a creative AI system that enhances human musician creativity by stimulating, inspiring, interacting, and cooperating in the flow of embodied live improvised music-making. Therefore, to build a robot driven by a Creative AI system, it must

1) continually improve by learning from humans and
2) create an effective and fulfilling human–robot interaction experience.

## The Project

My hypothesis to the research question posed before involved the design, development, and deployment of a robotic creative AI that would have a presence within the co-creativity of the flow of musicking and not be an AI zombie. This approach reinforces the personal understanding that when a musician enters the world of musicking, the “I” is coping in a very different world of concern than if they were walking down a street. In a sense, “I” becomes a different creature with a different set of priorities and concerns, outlooks, and sensorial inputs than my normal, human wakefulness. The technical and artistic solution for *EMR* focused on a robot that was first and foremost a coping entity in this specific world of concern (the flow of musicking).

The solution was to develop a system based on these three principles, expanded below:

1) Coping: *EMR* needed to cope in real time within the realm of musicking and be present as sound whose movements are embodied within such flow. This required a non-representational approach to how it related to the flow as the coping mechanisms needed to be open and dynamic enough to cooperate in any given musicking realm. Limiting the robot to a single representation of what musicking is, or might be, imposed onto the system by the human designer(s), would only work in a number of instances.
2) Creative AI dataset and experiential learning: these concepts needed to be designed from within the realm of musicking, prioritizing the phenomena of being inside this realm and capturing an essence of what it means to be embodied within the flow. The concept of experiential learning was designed to support this (discussed later).
3) Belief: the robot needs to believe in its view of the musicking world through limitations, embedded esthetics, and behavioral traits, even with glitches and bugs in the system.

From the human-centered artistic perspective, *EMR* needed to address the following:

- The robot was not an extension of the musician but should extend its creativity.
- The robot should not be an obedient dog or responsive insect jumping at my commands or impetus but a playful other.
- It should not operate as a simulation of play but as a stimulation of the human’s creativity.
- It is not a tool to enhance the human’s creativity but a being with presence in the world that they believe to be co-creating with them.
- It should prioritize emergence, surprise, and mischiefbut not expectation.

## Technical Solution

### [Not] The Solution

Before I describe my solution, I would like to describe what it is not using relatively well-known examples (NB is not the current state of the art). First, it is not an instrument-performing robot. For example, *TeoTronico* (2012) is a pianist-robot, designed and built by Matteo Suzzi. This robot plays the piano with dynamic control and articulation, moving 53 levers (described as fingers by Suzzi) with “great accuracy and speed” (Prosseda, 2014). In one example on YouTube, it plays a piece composed by Mozart, extremely well. It seems to have sensitivity about its performance, and even though the designers state that it uses MIDI files or be a “mirror pianist” (Prosseda, 2014), it does not sound like it is driven by a standard quantized MIDI file, so some form of human capture was used that stored a human performance as a MIDI file, which *TeoTronico* replayed. Its flow has been prepackaged and then regurgitated. Its sound is in the now, but its musicking is responding neither responding to the now nor to its environment. As such, to achieve the main aim of the *EMR* project, a technical and artistic approach such as this pianist-robot would fail as it simply could not cooperate with the human.

Second, it is not a goal-specific humanoid robot. For example, environmentally aware and goal-cognizant robots such as those being developed for the human–robot World Cup in 2050 (Robocup, 2015) are sophisticated robots employing the AI that make them aware of their world. In general, these systems use computer vision and sensors to navigate through this world; they have real-time awareness of here and now and are interacting with that in their goal to get the ball and score. The problem of using this kind of approach with the *EMR* project is in the nature of an embodied interaction. In musicking, the embodied relationships with other musicians are with the presence of the others as sound and not with them as human flesh. This relationship goes beyond relating to their physical presence and their movement, although it does play a part in varying degrees and at varying times, in my ongoing relationship-building process. So, an *EMR* needs to create relationships with human musicians through its *presence as sound* yet also has some physical presence and movement to inform this. Using these football robots as an analogy, it is not the physical movement of the robot moving toward the ball, or kicking, that creates the relationships required for this project but the relationship with the flow of the movement of the ball. As such, it is not the movement that incites the sound that is being related to in musicking but the presence of that sound in the flow.

### The Solution: *EMRv1*

The solution developed for *EMRv1* consists of the following three main concepts.

#### Coping

The design of *EMR* was informed by two early articles by the robot innovator Rodney Brooks, specifically *Intelligence without Reason* (Brooks, 1991) and *Intelligence without Representation* (Brooks, 1987). In these, he lays out the foundation of his approach to designing and building robots that are first and foremost able to cope and therefore adapt to a dynamically changing environment within the parameters of specific and multiple goals. This research eventually led to his robots being used for space and sea exploration, military and medical application, and the iRobot Roomba vacuum cleaner series. These Roombas are designed with *iAdapt* AI to be “creatures” that cope in a specific world of concern in real time. They neither have a model of representation of their world (such as building a 3D model of the space through computer vision and object analysis) nor do they make one as it goes about its business, but use goals and strategies to cope with whatever that world can throw at it (static furniture, steps, or chairs that get moved 1 day to the next).

Brooks’ foundational theories, and observations of my own Roomba, guided the developed for my *EMR* and generated this set of principles (adapted from Brooks (Brooks, 1987)):

- *EMR* must cope in an appropriate musical manner and in a timely fashion, with the dynamic shifts inside the musicking world;
- *EMR* should be robust to the dynamic environment of musicking; it should not fail to minor changes in the properties of the flow of musicking and should behave appropriately to its ongoing perception of the flow;
- *EMR* should maintain multiple goals, changing as required and adapting to its world by capitalizing on creative opportunity;
- *EMR* should do something in the world of musicking; “it should have some purpose in being” (Brooks, 1987).

#### Creative AI Dataset and Experiential Learning

This project innovated a different approach to computational learning that involved a human-in-the-loop and in-the-groove approach. This experiential learning (EL) approach trained the AI on the job and crucially inside the flow of embodied musicking. Furthermore, the EL process collaborated with a human musician who was equally learning about this new musicking system. This approach supported both the human and the AI to automatically learn and improve from experience.

**FIGURE 1:**
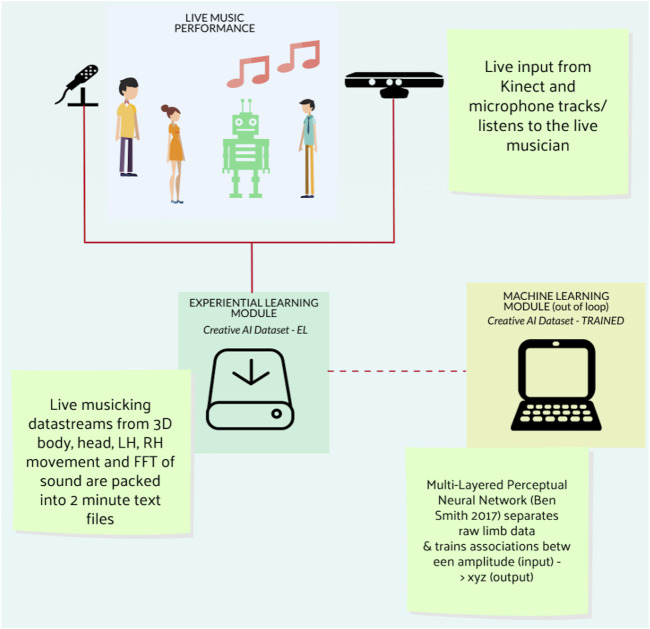
Experiential learning process. This image overviews the basic technical/data structure at play in EMRv1 and also is the foundation for further iterations of EMR.

The EL process (see Figure 1) focused on capturing the physical phenomena of an improvising human musician in the flow of creative musicking. The sensing mechanism used 3D depth tracking of the human musician’s body using a Kinect sensor (simply x, y, and z movement of both hands, body center, and head) and the fast Fourier Transfer (FFT) analysis of the live sound (fundamental frequency and amplitude).

**FIGURE 2:**
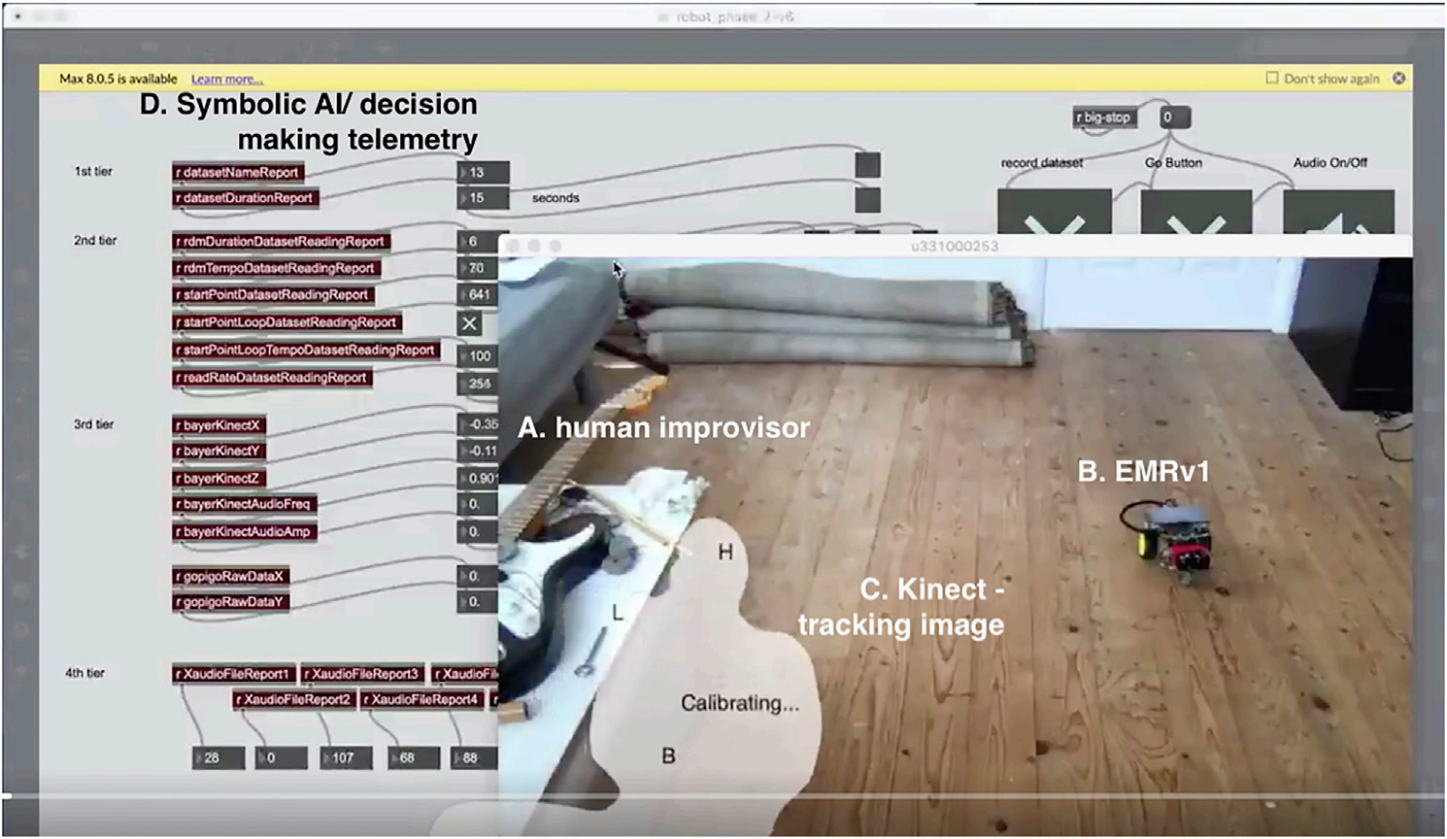
Image of a performance/training session between the author improvising on table-top electric guitar **(A)** and *EMRv1* **(B)**. The image collages the real-time tracking of the Kinect (the ghost image C.), and the data-logging page of Max/MSP patch **(D)**. A video of this session can be found online.^7^

The resulting dataset reflected the position and rotation of an embodied musicking body in motion together with the amplitude and frequency analysis of the actual sound made by such movement, without preserving the performer’s mass, musculature, melodic shape, or music. Thus, the embodied musicking movement is extracted from the performer’s body while they are making music; in a poetic sense, the dataset contains the meta-level DNA of musicking without the specifics or a representation of the music or the human. Recorded audio–video capture of the music performance would always anchor the dataset to a specific person and point in time, whereas the meta-level data could become the building blocks for the virtual composition (see Figure 2). Data phrases can be edited, treated, and repurposed by the robot’s AI again and again without the risk of repetition.

The initial process involved seeding the Creative AI dataset by capturing the live performance of an improvising musician. Once a small set had been generated, this musician then worked with the robot through a series of training sessions, with these new live data being added to the Creative AI dataset. The more they worked together, the more meta-level DNA of their shared creativity would be put back into the dataset system, thereby improving the AI’s knowledge base of the shared experience of embodied musicking.

The EL process was used in two ways: first, as a set of raw data that were called upon by the robot AI during a performance (see below), and second, as data for training the four neural networks. These separated the data into four body parts (head, body, right hand, and left hand) and trained a multilayered perceptron neural network using the body parts’ x, y, and z data to correlate with its amplitude for each line. Amplitude was decided as being the generator for the neural networks as the proposed application for *EMRv1* was non-idiomatic improvisation, and therefore, sonic impetus was determined to be a more appropriate factor.

The EL approach learns through an embodied interaction inside the flow of musicking. It utilized the meta-level DNA of its improvising partner—the human musician—and extracted elements from the dataset (any data randomly chosen by the system as it is all endowed with meta-level creativity) into its AI processing and then outputs the resultant sound as music. This EL process enhances the dataset through experience by its embodied coping inside the flow of musicking. The human musician perceives meaning in the robot’s musicking who in turn cooperates in the making of music (generally perceiving the relationship through one of the perspectives of creative cooperation discussed earlier) and responds with a creative solution through music. This is then captured using the sensing mechanisms and stored back into the Creative AI dataset. Thus, the cycle of EL continues to enhance and improve the dataset and enlarge the creative AI memory bank of deep and meaningful interactions between humans and robots which in turn forms the basis for future interactions.

#### Belief

It might seem odd to implement belief into the AI of a robot, given that this term usually refers to religious or spiritual faith, but it is the broader definition of this word that I am particularly interested. Specifically, *EMR* has an acceptance that something is true, or that it has trust or confidence in something from the perspective of the role its belief system plays in the behavior of EMR. I am not suggesting that *EMR* is sentient or has perception of the world but that the robot’s operational systems are embedded with structures that it can accept as guiding beliefs. They are:

1) *Movement behavior*: The robot’s movement operates within a behavioral system, designed to react openly to the dynamic sound world, and moves the wheels accordingly. The robot AI makes choices determined by whichever goal (listed before) is driving the wheels at any given point but within fixed parameters. The *Embodied Robot for Music* has freedom of choice to operate within such a field of response possibility. These are based on human preferences and outline a range of creative choices which have been determined over several decades. These are personal and subjective, and if these parameters were to be shifted or changed, then a different set of musicking characteristics would emerge. Within this structure, the robot has been embedded with a sense of esthetic that it can trust (believe to be true) and that the choices it makes are appropriate to it, co-creating inside the flow of musicking and unique to itself.
2) *Sound world*: The robot has a fixed sound library of roughly 1,000 short sounds, which were recorded through live improvisation, thereby embedding them with an essence of musicianship. These are triggered only when the wheels move. These are then either presented to the world in their raw state or treated in some way (time stretch, pitch shift, or both) using the Creative AI dataset as controlling parameters. The robot does not have the whole possible world of sounds, synthesis, and composition at its fingertips, but its sounds have a character and an esthetic basis which it can use to express its behavior and be unique to itself.
3) *Creative AI*: At the core of the creative AI, dataset is a world of embodied musicking captured through the EL process (described before) and through live interaction. These data are used to control every aspect of the AI, movement, sound production choice, and interaction goal. The dataset is also used to make choices about how the dataset is to be recalled and read by the algorithms (e.g., the read rate and ramp speed for each instance of wheel movement; discussed later). This means that the direct application of data into wheel movement and also the translations of that into sound object choice and therefore as music in the flow is imbued with the essence of embodied musicking that has been embedded in the core of the dataset. The version of the dataset in this application was a crude and small proof of concept. This has since been superseded by a larger project and a more comprehensive embodiment approach to the dataset.

But really these embedded belief structures are there so that the human musician can believe that the robot’s behavior and responses are truly emanating through musicking, and to draw attention to that fact, this robot is a valuable co-creative presence inside a shared flow. We all know that this robot is really an assembly of plastic and metal components together with a couple of motors and a processor. But because the human musician can trust it believes in certain things and has been embedded with a certain notion of its world of concern through concepts such as affectual response, its range of sonic choices, and its behavior, the human musician can believe in it as a co-creative collaborator inside musicking, which in turn can lead to deep and meaningful human-centered interactions.

### Technical Design

#### Hardware

The robot used in *EMRv1* was a Dexter *Go-Pi-Go* 1^2^ with a Raspberry Pi3 model B^3^ as the controlling computer. This system was used as the hardware was cheap, both the Dexter and the Pi had good online support and community forums, and there were plenty of ancillary peripherals available, such as cameras, which were equally cheap and supported. The *Go-Pi-Go* also came with an expanded version of the Raspian operating system (a Linux distribution) and included the libraries and dependencies to move the *Go-Pi-Go* completely with example scripts.

The robot was controlled remotely by the embodied AI in a black box system (discussed later) that broadcast movement parameters to the Pi. Onboard, the Pi was a simple script that received the transmitted parameters, translated them into wheel movement, and looked after the collision avoidance goal (below).

The embodied AI was built in *Max/MSP* ^4^ on a MacBook Pro and transmitted to the *Go-Pi-Go* using Open Sound Control (OSC)^5^ protocols over wireless. *Max/MSP* is a graphical programming language for multimedia development. It is quick and simple to use and is specifically designed for real-time editing and interactivity. This made software development quick and simple and facilitated rapid prototyping. *Max/MSP* also organizes threading and concurrency internally.

#### *EMRv1* Subsumption Architecture

The technical design of *EMRv1* was influenced by robotic subsumption architecture. This is a control architecture innovated by Rodney Brooks as an alternative to traditional AI, or GOFAI. Instead of guiding the robotic behavior by symbolic mental representations of the world, subsumption architecture “is a parallel and distributed computation formalism for connecting sensors to actuators in robots. A traditional way of describing these connections would be to say the subsumption architecture provides a way of writing intelligent control programs for mobile robots” (Brooks, 1986).

The subsumption architecture was designed to support multiple goals. These were (in order of priority) given as follows:

1) *Self-preservation*: The robot must avoid obstacles and not crash into the other musician or fall off the stage.
2) *Instinctual behavior*: If left alone, the robot would make music. This was driven by the Creative AI dataset (discussed before), which operated as its DNA of musicking creativity.
3) *Dynamic interaction*: The robot can, in certain conditions, be affected by the sound of the live musician. Using a process of simulated affect linking, the Creative AI could leap between related, abstracted, or unexpected datasets. Metaphorically, the robot’s internal trains of thought would be triggered by phrasing (short-term temporal limits) and the dynamic impetus of the human.

A critical feature of the design of *EMR* is that each of these goals directly moves the wheels. It was essential that each goal is not part of an elaborate, logically flowing representation of a thought process, mimicking some kind of mind. As such, the overall design of the robotic system was modular, with each system directly accessing the wheels when in operation.

The overall modular design of the data flow is given as follows:

1) Live data sensors
2) Data wrangler
3) Affect mixing
4) Smoothing and deviation
5) Wheels move. Make sound

Although this may appear to be a linear flow based on a hierarchy, the subsumption design is embedded in module 2 *Data wrangler module*. With module 3 *Affect mixing*, enhancing the non-hierarchical approach. The code for *EMRv.1* is freely available as open-source on GitHub.^6^

The design of each module is as follows:

1) Live data sensors

This module coordinates and streams the live sensor data to various modules across the system. The live input consists of a line input signal from the collaborating musician (human or robot), and a stream of OSC data from the Kinect (x, y, and z coordinates of the head, body, left hand, and right hand). The audio from the line input was analyzed for dominant (fundamental) frequency and amplitude. These were then concatenated together as a series of lists and stored as dataset files for immediate use and access in later training sessions, and performances. The sample rate was flexible and was triggered by the incoming Kinect data. Table 1 illustrates how these were saved as .csv files.

**TABLE 1:** Example of the Creative AI dataset.

Id	Limb	X	Y	z	Freq	Amp
35	/Hand_Left	−0.31917	−0.295,487	1.376,182	161.538,467	0.322,659
36	/Hand_Right	−0.264,689	−0.213,074	1.28068	161.538,467	0.322,659
37	/Body	−0.397,107	0.106,659	1.222,754	161.538,467	0.322,659
38	/Head	−0.246,853	0.314,369	1.072035	161.538,467	0.166,799
39	/Hand_Left	−0.372,583	−0.077763	1.275,277	161.538,467	0.166,799
40	/Hand_Right	−0.256,269	−0.215,644	1.274,499	161.538,467	0.166,799
41	/Body	−0.387,607	0.108,567	1.23542	161.538,467	0.166,799
42	/Head	−0.239,018	0.316,554	1.083863	114.248,703	0.11613
43	/Hand_Left	−0.375,174	−0.039334	1.263,249	114.248,703	0.11613
44	/Hand_Right	−0.248,108	−0.212,755	1.270,422	114.248,703	0.11613
45	/Body	−0.365,129	0.119,221	1.260,812	114.248,703	0.11613
46	/Head	−0.23085	0.319,204	1.095646	31.987,429	0.131,989
47	/Hand_Left	−0.396,978	0.060928	1.210,986	31.987,429	0.131,989
48	/Hand_Right	−0.223,919	−0.181,981	1.253,668	31.987,429	0.131,989
49	/Body	−0.356,796	0.122,125	1.268,893	31.987,429	0.131,989
50	/Head	−0.227,154	0.319,129	1.099726	31.987,429	0.131,989
51	/Hand_Left	−0.456,557	0.253,543	1.086275	31.987,429	0.131,989
52	/Hand_Right	−0.208,468	−0.130,853	1.233,942	31.987,429	0.10922
53	/Body	−0.342,327	0.127,401	1.279,092	31.987,429	0.10922
54	/Head	−0.227,208	0.318,383	1.099777	31.987,429	0.10922
55	/Hand_Left	−0.323,183	−0.030516	1.199,504	31.987,429	0.10922
56	/Hand_Right	−0.173,354	−0.011939	1.149,591	31.987,429	0.10922
57	/Body	−0.332,003	0.131,065	1.283,651	31.987,429	0.10922

The operational processes of *1. live data sensors* module were given as follows:

i) capture the live sensor data and concatenate it into data lists;
ii) package the data lists as .csv files in the dataset local directory;
iii) stream each of the fields to other modules for use in real-time decision-making processes.
2) Data wrangler

This module generated the metaphorical trains of thought for *EMRv1* in the following two ways:

1) querying and reading from the files stored in the Creative AI dataset directory
2) generating outputs from four neural networks trained on the Creative AI dataset (discussed before).

The basic process for the querying and reading from the files stored in the Creative AI dataset directory was designed to symbolically represent the shifting nature of trains of thought as proposed by (Gelertner, 1994). The symbolic process was constructed as follows:

i) Choose a dataset file from the directory for a random duration (6–26 s)
ii) If an affect signal is received (see below), change the file immediately [goto 1]
iii) Choose a random line to start reading from the dataset file
iv) Start reading from this line for the random duration (3–13 s)
v) Read at a random procession rate (300–1,300 ms)
vi) Loop if triggered
vii) Parse and smooth all fields from the dataset as individual data atoms and send them to next module

The basic process for generating outputs from the four neural networks trained on the Creative AI dataset was triggered by the amplitude data received from three sources: 1) the live audio input (after FFT separation), 2) from the querying process before, and 3) from a short-term memory buffer that looped and recorded the live improvisation, and randomly read the audio from any point. Each of these was mixed and routed into each of the four neural networks, from which was generated x, y, and z data, which were streamed to the next module.

3) Affect mixing

This module received all the data streams from the dataset query, parsing process, and the neural networks and mixed them into the following two outputs: left wheel data and right wheel data. The mixing was controlled by a special process designed to symbolically represent affect and affect-linking (Gelertner, 1994) of a musician. In Vear 2019, I defined affect as “the mind’s connecting response between sensorial input of external events with the internal perception of causation such as emotion or feeling, through time.” This module translated this definition symbolically, the streams of amplitude data from the live input, the dataset parsing, and a randomly generated “drunk walk,” would be used to trigger 1) local changes in the module such as mix and 2) global conditions such as dataset file selection. The basic process was given as follows:

i) randomly switch between input streams (1–4 s, or with a loud affect trigger)
ii) if amplitude is <40%, do nothing
iii) else if amplitude is between 41 and 80%, trigger a new mix (see below)
iv) else if amplitude is >80%, trigger condition changes across the architecture (new mix, new file read, restart reading rate, change smoothing rate, and change audio read in following modules)

The mix function randomly selected which of the incoming data streams (x, y, and z from dataset read, x, y, and z, from live Kinect, x, y, and z from the neural network prediction) to be the output to the following module for the wheel movement. It was desirable that this involved multiple elements from these incoming streams being merged, metaphorically fusing different trains of thought into a single output.

4) Smoothing and deviation

The final stage in the dataflow process smoothed the output for each wheel using random slide properties of 15–450 ms. This would introduce a sense of push and pull in the final wheel response and sound generation, and like the other random processes were symbolic and metaphorical representations of rhythm and phrase generation. The last part of this process looked for deviations in changing data using a delta change function !(n - n-1). This was then sent to the wheel module.

5) Wheels move. Make sound

The left-wheel and right-wheel data outputs from the aforementioned module were rescaled and then sent via OSC and wireless to the *Go-Pi-Go* robot, which parsed them and moved the wheels. Simultaneously, these data were sent to the *Make Sound* module, which made independent sounds for each wheel. The data were rescaled between 0 and 1,177 so that it would trigger one of the minute samples held in its belief system (discussed before). These samples were then projected from speakers attached to the laptop.

## Discussion

The debate on whether intentionality is needed for creativity is still ongoing in the literature (Paul and Kaufman, 2014). *EMRv1* does not have a module in its subsumption architecture that deals with intentionality. Yet, I perceived moments of it intentionally responding to musicking, and also not responding to keys and triggers such as sonic impetus. This malleability in its response was intentional as I wanted to be surprised by what it did and did not respond to, in the same way that another human musician can choose to react to a musicking moment or not. This approach acts as a metaphor for my surprise when in musicking I make an unexpected response. In these moments (which happen regularly), I did not intend to respond, but something inside my being emerged. I cannot explain this, but I am aware of it, and the possibility of this is embedded in the experiential learning process and the Creative AI dataset with the symbolic AI making space for this to happen, or not.

It could be argued that this AI system is a passive passenger along the flow of musicking privileging the human musician as the central driver for all musicking decisions. And this is true, on a superficial level. All perceived interactions are from the human perspective, who in turn responds with a human-orientated decision. However, the embodied presence of the robot (in contrast with the presence of only a computer/non-anthropomorphic artificial system) did influence my human responses as I recognized its movements as being in the groove, due to them being based on my movements. This sense of familiarity with the movement (which in turn begat the sound) contributed to a sense that this *EMRv1* was inside its flow. This led to a sense of “meaning” as I felt that we were journeying together through a shared flow. Any points where I felt that the relationship was *concurrent*, *collaborative,* or *co-creative* further reinforced this, leading to a heightened sense of togetherness. I should add here that if *EMRv1* was left to perform a solo, it would do so without the need for human intervention. This was part of its “purpose of being.” Similarly, when I placed two robots together, they performed a duet (Patabots, 2019a) (Patabots, 2019b).

It is a limiting factor here that due to lockdown and COVID pandemic restrictions, this research was unable to engage with other musicians and so remains anecdotal. But there is something in the way that *EMRv1* responds inside musicking that brought me closer to the improvisational relationships I have with other musicians. This is due to the goals and purpose embedded into the AI and robotic architecture of *EMRv1* being loose, and focused on surprise and novelty, as opposed to some elaborate mind-based model.

For me, this type of creativity happens inside a system that propagates principles of play and invention but is also bound by limits and parameters. Even the notion of free improvisation is bound to an individual’s imagination and technique. Notions of “meaning” and “purpose” are therefore bound to enabling this system to operate within such parameters and limits. Meaning is the preserve of the human who recognizes that the system is in the flow, believes that its system is playing and inventing, and responds with creative playfulness. The robot AI has purpose, which is to play and invent within this system. Together, these create a system that can lead to emergent creativity. But this is not guaranteed; but neither is it guaranteed between human–human improvised musicking.

The consequence of this study is that it could signify a fruitful way forward of interpreting the concept of natural and artificial co-creativity. Considering playful creativity in AI as a defined system with a purpose, rather than a set of ingredients might unlock small-c creative projects. However, this also opens these applications to moments of failure as the system cannot be guaranteed to be creative all the time due to its inbuilt freedom, the integrity of the dataset, and the reliance of the human to comprehend what is understood as “meaning” in the flow.

## Conclusion

Using the principles outlined earlier, the *EMRv1* project has created a co-creative system that responds to the interaction with a human musician through a cyclical relational process. It is important to note that the interaction with the musician begets movement as its primary goal for musicking and that this movement is embedded with the essence of embodied musicking because of the experiential learning process. Following this, the movement begets sound, which begets music such that all relationships between humans and AI are informed by phenomenon data captured within the embodied flow of music-making: either from the Creative AI dataset or through live interaction.

The subsumption architecture appeared to create a solution for an intelligent coping that followed the principles of the project (listed before). But due to COVID lockdown restrictions and budgetary factors, the testing of *EMRv1* was restricted to the author. However, these improvisations were presented on multiple occasions in front of the general public and peers, with encouraging responses and requests to try it out.

The design of *EMR* supported simple changes to its internal belief system that resulted in a change of behavior and esthetics. For example, swapping the source audio files for another set made the robot sound different. Changing some of its internal random parameters, especially in module 2 *Data* w*rangler* and module 4 *Affect mixing*, had a significant effect on its internal rhythmic and phrasing structures, thereby responding to the live improvisation with a different feel.

The ultimate goal of this research is *not* to find solutions to replace human creativity but to enhance it and move it forward into discoveries. In short, this research is seeking to find experiences like those emergent through DeepMind and Alpha Go’s interaction with the professional Go players. In the 2019 film (AlphaGo, 2017), several of these professionals reflected that when they played with AlphaGo, they “see the world different […] Maybe it’s beautiful,” and “like move 37, something beautiful occurred there”; “in a broad sense move 37 begat move 78 begat a new attitude, a new way of seeing the game he improved through this machine, his humanness was expanded after playing this inanimate creation” (AlphaGo, 2017).

**FIGURE 3:**
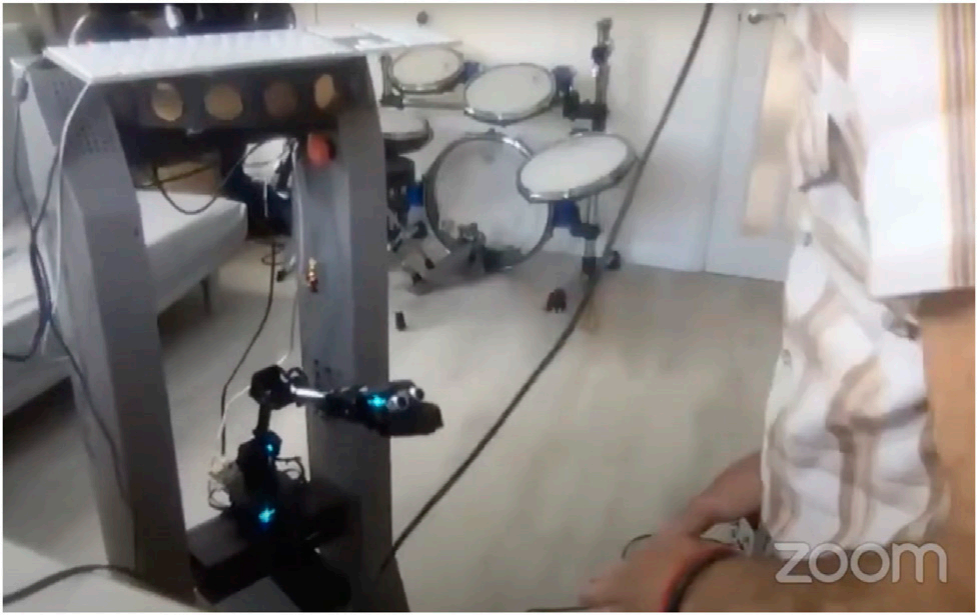
A screengrab from an online performance of EMRv4 and the author as part of the Art-AI festival 2021.

I am hopeful, given the current trajectory and generation (v4) that this foundational work outlined in this article has proven to be a viable solution for such emergent creativity between musicking humans and robots. But as we are all still managing the COVID pandemic, and the limitation of face-to-face research, it may be a while before I am able to test EMR with another unbiased collaborator. However, as EMR grows with each iteration, the feeling of stimulated relationships in musicking grows. At a recent public talk/performance for the Art-AI festival 2021, I gave a demo performance of EMRv4^8^ and I playing together (see Figure 3). It is interesting to note how the movement begets sounds and how the movement emits a sense of musicking, regardless of the sound produced [https://www.youtube.com/watch?v=LryGSo7MK74&t=3370s]. But, as mentioned before, this phenomenon needs a considerable amount of further testing and validation and so remains only a mere hopeful conclusion.

## Data Availability

The datasets presented in this study can be found in online repositories. The names of the repository/repositories and accession number(s) can be found below: github.com/craigvear.

## Footnotes

2
*Go-Pi-Go*. Available at: https://www.dexterindustries.com/gopigo3/ (Accessed 2020/10/23).

3 Raspberry Pi. Available at: https://www.raspberrypi.org/products/raspberry-pi-3-model-b/?resellerType=home (Accessed 2020/10/23).

4 Max/MSP. Available at: https://cycling74.com/ (Accessed 2020/10/23).

5 Open Sound Control. Available at: http://opensoundcontrol.org/introduction-osc (Accessed 2020/10/23).

6 GitHub. Craig Vear. Available at: https://github.com/craigvear/Seven_Pleasures_of_Pris (Accessed 2020/10/23).

7 Twitter. Craig Vear. Available at: https://twitter.com/i/status/1123569268673716233 (Accessed 2020/10/23).

8 EMR v4 has now been migrated to Python and uses a hive of neural networks trained using the TensorFlow library, cooked using a dataset from the “embodied musicking dataset” repository. Available at: https://github.com/Creative-AI-Research-Group/embodiedMusickingDataset/tree/master/dataset.
